# Supplementary material for: Structural basis for reduced ribosomal A-site fidelity in response to P-site codon–anticodon mismatches
Source: J Biol Chem. 2023 Mar 15;299(4):104608. doi: 10.1016/j.jbc.2023.104608 (PMC10140155; doi:10.1016/j.jbc.2023.104608)
Supplement: Supporting Figures S1–S4 and Table S1 [file mmc1.docx]

**Structural basis for reduced ribosomal A-site fidelity in response to P-site codon-anticodon mismatches**

Ha An Nguyen^1,2^, Eric D. Hoffer^1,2,3^, Crystal E. Fagan^1,2,3^,

Tatsuya Maehigashi^1,2^ and Christine M. Dunham^1,2*^

^1^Department of Chemistry, Emory University, Atlanta, GA, USA

^2^Emory Antibiotic Resistance Center (ARC), Emory University, Atlanta, GA 30322, USA

^3^Biochemistry, Cell and Developmental Biology Graduate Program, Emory University,

Atlanta, GA, USA

*Contact: Christine M. Dunham, christine.m.dunham@emory.edu

**Supplementary data file contains**

**Figures S1-4**

**Table S1**

**Data deposition:** X-ray crystallography, atomic coordinates, and structure factors have been deposited in the Protein Data Bank, www.pdb.org (PDB codes 8FOM, 8FON)

**Key words:** ribosome, miscoding, near cognate, mRNA, tRNA, fidelity, translation

# **Supplementary Figures**

**
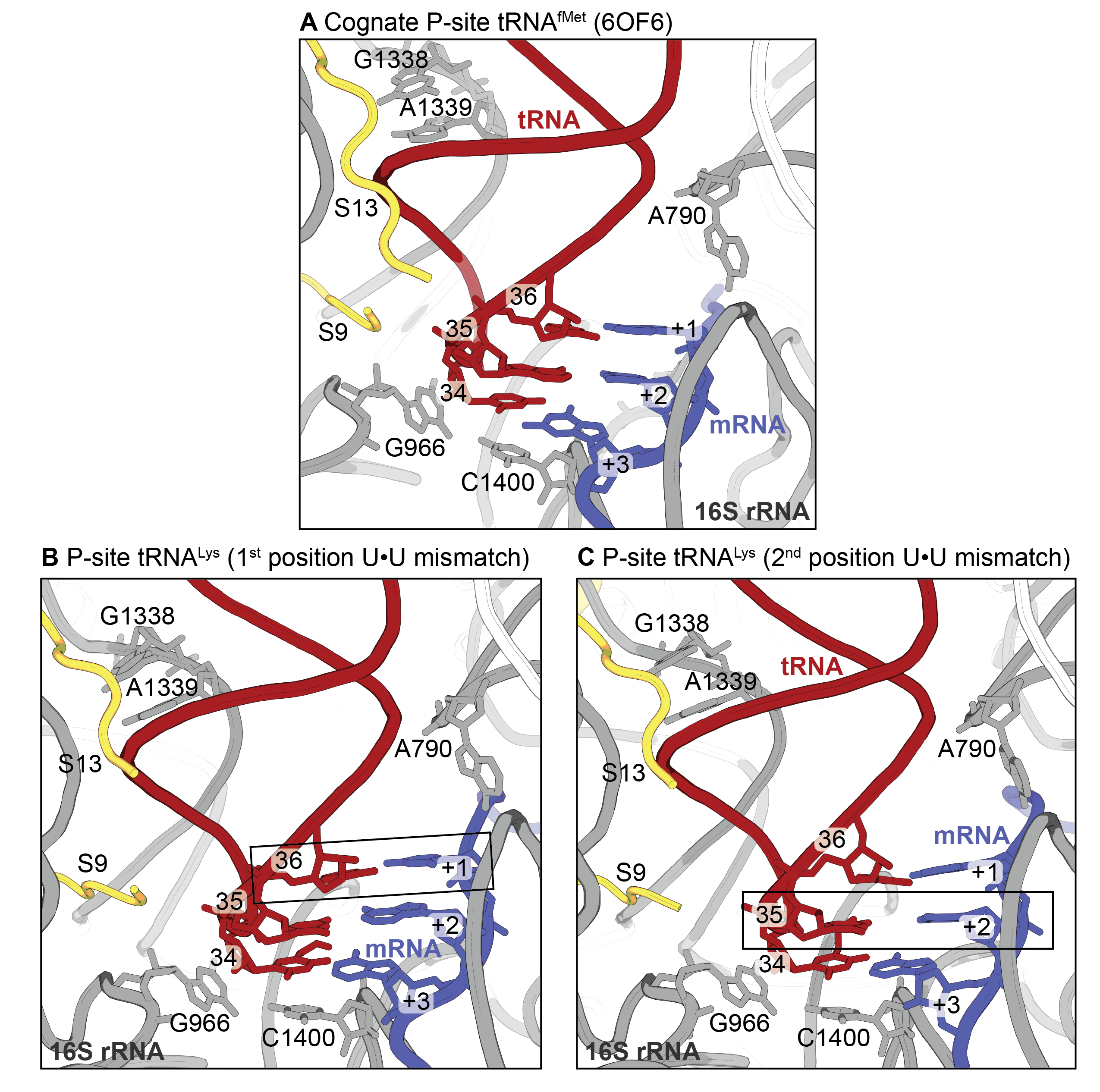
**

**Figure S1. The ribosome does not recognize the mismatches in the P-site codon-anticodon interaction.** The ribosomal environment of a cognate P-site tRNA^fMet^ bound to the start codon in the P site (PDB code 6OF6) (A) is similar to the two structures containing P-site mismatches at the first (B) or second position (C). The three base pairs of the codon-anticodon interaction are not extensively probed in the P site, and the P site is not known to have proofreading capability like that of the A site. In all three structures shown here, the P-site rRNA nucleotides and ribosomal proteins (r-proteins) critical for tRNA binding and translocation do not have any structural response to the mismatch in the first (B) and second position (C) of the codon-anticodon interaction: 16S rRNA nucleotides G966 and C1400 pack against the third anticodon-codon interaction similarly in all three structures, A790 is in the same conformation, G1338 and A1339 grips the stem of the anticodon loop, and r-proteins uS9 and uS13 tails form the tRNA binding site in the same way as when a correct tRNA is bound (A).


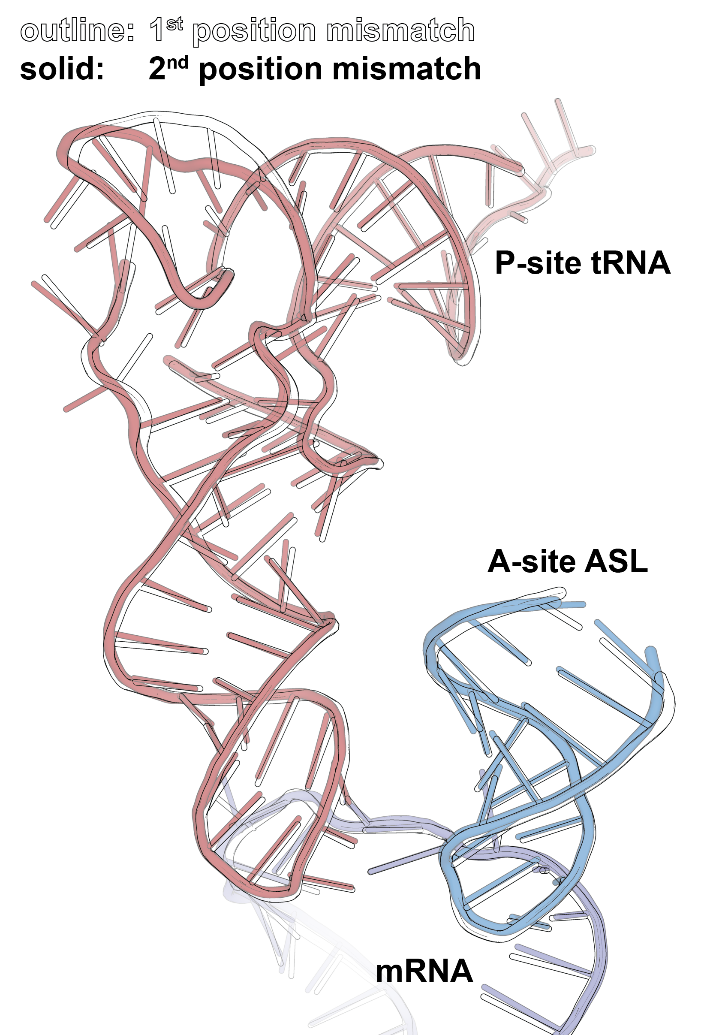


**Figure S2**. **Overlay of the P-site tRNAs of two structures in this study shows similar tRNA conformations**. The tRNA^Lys^ bound to the 1^st^ position mismatch (in black outline) looks almost identical to when it is bound to the 2^nd^ position mismatch (in red). The A-site ASL and the mRNA are also similar, with the overall all-atom root-mean-square deviation (RMSD) of the P-site tRNA, A-site ASL, and the mRNA being 0.357 Å.


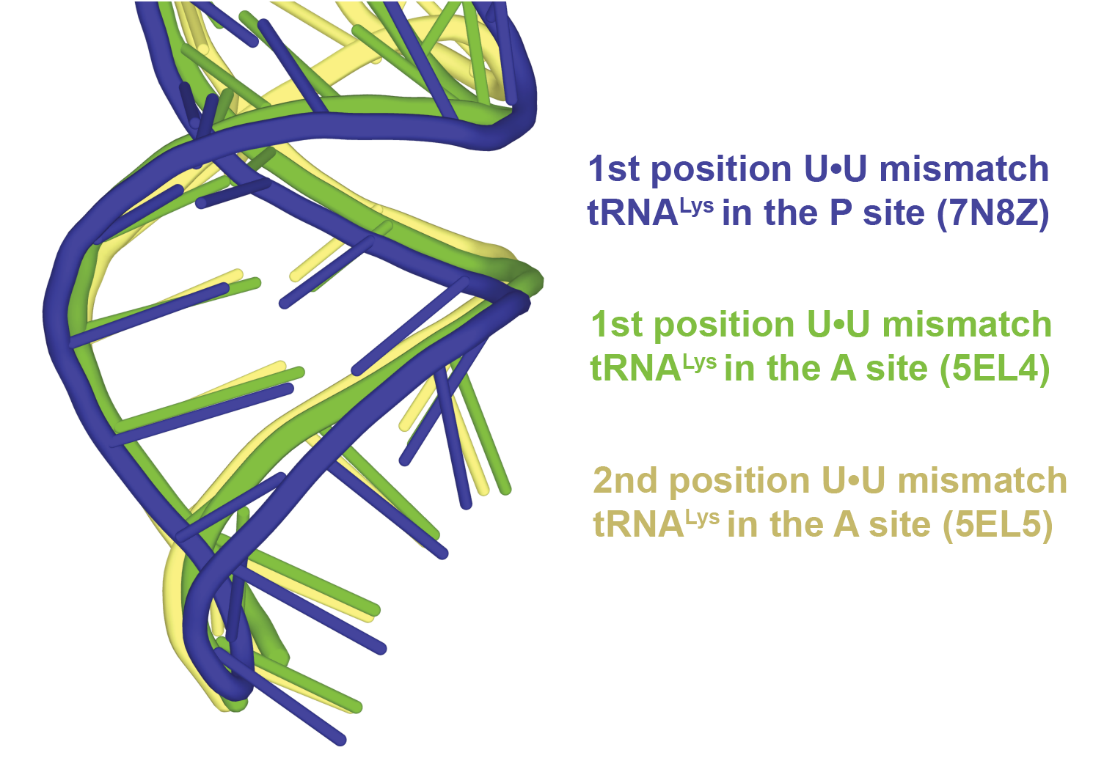


**Figure S3. Comparison of tRNA^Lys^-mRNA codon mismatches.** The anticodon stem loop conformation of the P-site tRNA^Lys^ 1^st^ position mismatch is similar as compared to the A-site tRNA^Lys^ with a 1^st^ (green) and 2^nd^ (yellow) position U•U mismatches (Rozov et al. 2016).


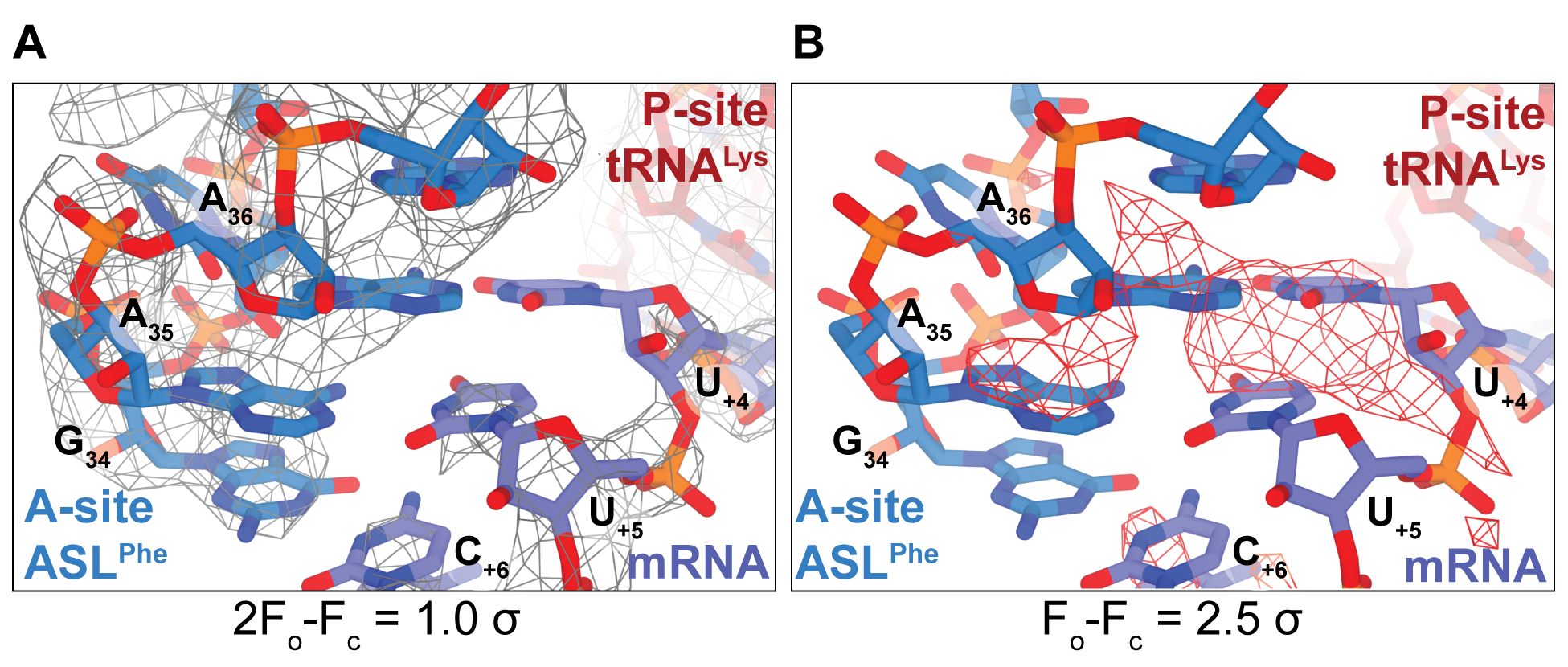


**Figure S4. Electron density maps after refinement of the structure containing the 2^nd^ position P-site mismatch when U_+4_ and A_36_ are modeled to form a base pair in the A site.** A. The gray mesh overlay represents the 2F_o_-F_c_ map contoured at 1.0 σ showing lack of density supporting the U_+4_ and A_36_ forming a Watson-Crick base pair. B. The red mesh overlay represents the F_o_-F_c_ map contoured at 2.5 σ showing strong negative difference density for the modeled base pair.

**Table S1. RNA sequences used in this study.** For the mRNA sequences, the AUG start codon is located in the E site, the bolded codon is located in the P site and the UUC Phe codon is located in the A site. For ASL^Phe^, the bolded nucleotides represent its GAA anticodon.

| mRNA P-site UAA codon | 5’- GGC AAG GAG GUA GGG AUG **UAA** UUC AAA -3’ |
| --- | --- |
| mRNA P-site AUA codon | 5’- GGC AAG GAG GUA GGG AUG **AUA** UUC AAA -3’ |
| ASL^Phe^ (GAA anticodon) | 5’- GGG GAU U**GA A**AA UCC CC -3' |

**REFERENCES**

Rozov A, Demeshkina N, Khusainov I, Westhof E, Yusupov M, Yusupova G. 2016. Novel base-pairing interactions at the tRNA wobble position crucial for accurate reading of the genetic code. *Nature Communications* **7**: 10457.
